# “Promote locally led initiatives to fight female genital mutilation/cutting (FGM/C)” lessons from anti-FGM/C advocates in rural Kenya

**DOI:** 10.1186/s12978-020-0884-5

**Published:** 2020-02-28

**Authors:** Purity Mwendwa, Naomi Mutea, Mary Joy Kaimuri, Aoife De Brún, Thilo Kroll

**Affiliations:** 1 School of Nursing, Meru University of Science and Technology, Meru, Kenya; 2 School of Nursing, Midwifery and Health Systems, University College Dublin, Dublin, Ireland

**Keywords:** Female genital mutilation/cutting, Anti-FGM/C advocates, Meru, Kenya

## Abstract

**Background:**

Female Genital Mutilation/cutting (FGM/C) is a tradition rooted in culture and involves the partial or total removal or other injury to the female genital organs for non-medical reasons. In Kenya, initiatives to abandon the practice have included ‘alternative’ ritualistic programmes (ARPs) combined with intensive community sensitisation about FGM/C to achieve attitudinal and behavioural changes. While there are indications of the effectiveness of these interventions, FGM/C continues to be practiced within certain groups in Kenya. This study explored the views of anti-FGM/C advocates on the barriers and facilitators to tackling FGM/C within the Meru community in Kenya.

**Methods:**

Data were obtained using 4 Focus Groups (FGs) with 30 anti-FGM/C advocates from Tigania East and West in Meru county. Thematic framework analysis guided the analysis based on four main questions: 1) How has the cultural meaning of FGM/C evolved over time? 2) What are the perceptions in relation to the effectiveness of anti-FGM/C interventions? 3) How effective are interventions and campaigns to end FGM/C in Meru county? 4) What actions are perceived as the most likely to bring about change?

**Results:**

There has been a substantial shift in the culture of FGM/C and the number of families carrying out the practice in Meru county has decreased in recent years. Participants noted five actions likely to bring about change; 1) reviving and supporting ARPs, 2) encouraging fathers’ involvement in the upbringing of their daughters,3) inclusion of the topic of FGM/C in the current education curriculum and public fora, 4) strengthening the community policing strategy -*Nyumba Kumi*, 5) and setting up community centers for orphans.

**Conclusion:**

Our findings demonstrate the significance of locally led initiatives to fight FGM/C. It also became clear that change would have to start at the family level with parents, particularly fathers, taking on a more active role in the lives of their daughters. Providing education about FGM/C to communities, particularly young men coupled with keeping girls in school appeared to be some of the most effective ways of fighting FGM/C. At the community level, the church became particularly crucial in challenging the practice of FGM/C.

## Plain English summary

Female Genital Mutilation/cutting (FGM/C) involves the cutting or removal of female genital organs for nonmedical reasons. FGM/C is a common practice among some communities in Kenya where anti-FGM/C advocates continue to play an important role in encouraging change. The aim of this study was to explore the views of anti-FGM/C advocates on the barriers and facilitators to tackling FGM/C within the Meru community in Kenya.

We conducted 4 Focus Groups (FGs) discussions with 30 anti-FGM/C advocates (men and women) from two rural sub-counties in Meru, Kenya. We asked them questions about the changing culture in the practice of FGM/C, the role played by men and women in the continuation or ending of the practice, the impact of interventions and campaigns aimed at ending FGM/C and what they considered the most effective actions that would bring about sustained about change.

There seems to be a considerable shift in the culture of FGM/C and the number of families carrying out the practice in the two sub-counties has drastically decreased. Education, particularly for girls, interventions led by religious groups, and ARPs led by anti-FGM/C advocates seem to have played a crucial role in this change. Anti-FGM/C advocates acknowledged the need to promote locally grown and led initiatives including reviving and supporting ARPs and building on existing initiatives such as the community policing strategy -*Nyumba Kumi.* Encouraging fathers to get actively involved in the upbringing of their daughters, as well as including the topic of FGM/C in the current education curriculum were viewed as potentially crucial to realizing change in the practice of FGM/C in Meru county.

## Background

Female Genital Mutilation/cutting (FGM/C) is a tradition rooted in culture and involves the procedures that intentionally alter or cause injury to the female genital organs for non-medical reasons [1]. It is estimated that at least 200 million girls and women in 30 countries have been subjected to the practice [2]. Over the past 40 years interest in curtailing the practice has intensified [3] and several awareness and abandonment approaches have been deployed with the aim of influencing behaviour change [4, 5]. While there has been an overall decline of FGM/C current progress is not sufficient to keep up with increasing population growth and the number of girls and women undergoing FGM/C is likely to rise significantly over the next 15 years [2].

Initiatives to abandon the practice of FGM/C in sub-Saharan Africa (SSA) date back to the colonial times. Such efforts were motivated by different understandings and employed a variety of approaches, including those based on human rights frameworks, a health risk approach, training health workers as change agents, and the use of comprehensive social development approaches [3]. In some countries in SSA evidence points to the pivotal role of ‘alternative’ ritualistic programmes (ARPs) combined with intensive community sensitisation about FGM/C to achieve attitudinal and behavioural changes [6]. Yet while ARPs are considered a catalyst for change, outcomes within a community vary and are dependent on the context, time and the manner in which the intervention is implemented) [7].

While the prevalence of FGM/C in Kenya in recent years is relatively low (21%), FGM/C is distributed variably throughout the country, with prevalence ranging from 1% to 98% across provinces [8]. In Kenya, efforts to abandon FGM/C were pioneered by Protestant Christian missionaries [9]. Over the years, other organizations including Maendeleo ya Wanawake Organization (MYWO), a community based women’s organisation, joined the fight against FGM/C by using communication for social change initiatives and ARPs that embrace positive traditional values and exclude FGM/C [6]. There are indications of the effectiveness of some of these interventions, particularly community-led approaches that have targeted high prevalence areas like Tigania and Igembe in Meru county in Kenya [10].

Meru is one of Kenya’s 47 counties, and anti-FGM/C campaigns date back to the ‘Ngaitaana’ (‘To circumcise oneself’) generation of the early 1960s when the colonial government outlawed the practice but girls from Meru North resisted the move and demanded the cut [9]. Once the practice was outlawed in the country in 2011, it is believed that the practice went underground prompting non- governmental organisations (NGOs) and churches to come up with ARPs dubbed ‘Ntanira na mugambo’ (circumcision with words) to empower young girls to resist the cut in Meru county [10]. While Meru is not one of the high prevalent counties, (Meru 36.7% compared to the Somali community 93.6%, [8] the county has seen a long record of anti-FGM/C activities, particularly the ARPs. These interventions have been deployed through intensive media outreach, the involvement of policymakers, anti-FGM/C advocates, national religious leaders, leaders of civil society organizations and international development agencies, such as PATH (Program for Appropriate Technology in Health). And while such initiatives have served to raise awareness in an effort to change beliefs, attitudes, behaviours and practices related to FGM/C, recent media reports show that FGM/C continues to be practiced in this region. In December 2018, a 14 year old girl died following complications related to FGM/C [11], while other reports indicate that the war against FGM/C in this county is yet to be won as the practice continues to be performed secretly [12]. One explanation may be that interventions to halt the practice tend to be isolated and uncoordinated [13] and some of the interventions do not aim to change the cause of the practice - the “mental map” [14]. Research on which interventions are most influential is essential to efficiently allocate limited resources from the offset [14]. The aim of this exploratory study was to examine the experiences and barriers as well as facilitators to tackle FGM/C in Kenya from the perspectives on anti-FGM/C advocates. Specific objectives included;

1. To determine how the cultural meaning of FGM/C has evolved over time
2. To assess men’s and women’s perceptions regarding the practice of FGM/C
3. To understand the effectiveness of interventions designed to fight FGM/C
4. To identify potentially effective and sustainable interventions

## Method

This qualitative exploratory study employed FGs with anti-FGM/C advocates from two rural sub-counties in Meru, Kenya, where according to recent reports, FGM/C continues to be practiced [11, 12, 15]. In Meru county, efforts to abandon FGM/C have been led by anti-FGM/C advocates, particularly through ARPs which consist of a series of activities replacing FGM/C with non-harmful traditional rituals highlighting girls’ initiation into adulthood. Anti-FGM/C advocates have been involved in education programs for families, teaching them about the dangers of FGM/C and challenging the stigmas around uncut girls, who are traditionally ostracized from society and find it hard to marry. This group is therefore potentially knowledgeable about the gaps and opportunities for designing successful anti-FGM/C initiatives.

FGs were considered the most appropriate technique to collect data on the topic as group interaction would encourage respondents to explore and clarify individual and shared perspectives [16]. Purposive and snowball sampling was used to select participants. Participant recruitment occurred through a community-based group engaged in anti-FGM/C campaigns in Meru county. Individuals were eligible to take part in the study if they were previously or currently engaged in anti-FGM/C campaigns in the two sub-counties. Once initial contact was made with a group of anti- FGM/C advocates, snowball sampling was used to identify other prospective participants. None of the participants invited to take part declined and in total, 30 participants were recruited and each of the FGs included six to nine participants. Given the sensitivity of the topic, the decision of where to conduct interviews was discussed with participants to determine the most appropriate (and their preferred) environment. Three FGs were conducted on church premises and one in a sub-county meeting room.

## Data collection

FGs were moderated by the first author (PM). The FGs began by asking broad questions about the topic (for example what meaning FGM/C carries in the community and whether or how this meaning has changed over time), before asking the focal questions. Among the topics explored in the interview guide included 1) examining the reasons why the practice continues or decreases 2) appraising the effectiveness of strategies to curb the practice 3) Identifying new strategies and points of actions to curb the practice of FGM/C. Before the FGs commenced, participants were provided with written consent forms and given time to read information sheets, reflect on their participation and ask the researcher questions. They were then asked to sign the consent form to indicate their agreement to take part in the study. None of the participants approached declined to participate. As the first author speaks the local language, FGs were conducted in the local language. A total of four FGs were conducted, with each FG lasting between 45 and 60 min. Participants were reimbursed for their time and travel expenses. FGs were audio-recorded, transcribed and translated to English by the first author.

## Data management and analysis

To protect the identity of participants and maintain the confidentiality of the data collected, study codes were used on data documents. The paper field notes, and transcripts of interviews were anonymised and no names or any identifying details were included. We ensured that transcripts of audio recording, had no reference to the identity of the person and during transcription, any identifiers were removed or aggregated by using pseudonyms, or vaguer descriptors. To protect data during travel between research locations, the data were encrypted. All data were stored on password-protected computers and only the primary researchers had access to the data.

Data were analysed thematically [17] with themes derived from the data. Thematic framework analysis was designed to guide the analysis and two researchers (PM and ADB) coded the data. This qualitative method of analysis was most suited for this study as the prime concern was to describe and interpret what is happening in a particular setting to generate recommendations or outcomes within a limited time period in regards to a given policy or policy issues [18]. The analysis was based on four main questions: 1) How has the cultural meaning of FGM/C evolved over time? 2) What are the perceptions in relation to the effectiveness of FGM/C interventions? 3) How effective are interventions and campaigns to end FGM/C in Meru county? 4) What actions are perceived as the most likely to bring about change? An initial coding framework was devised based on findings from the literature and guided by the structure and line of questioning in the FGs. This apriori framework was subsequently refined in the inductive coding process.

## Results

### Participant characteristics

Table 1 contains the participant characteristics in the sample.

**Table 1:** Social-demographic characteristics

	n	%
Location		
Tigania West, Meru	21	70
Tigania East, Meru	9	30
Gender		
Male	14	47
Female	16	53
Age		
29–44	5	16
45–60	16	53
61–66	4	13
67–82	5	16
Marital status		
Married	28	93
Widowed	2	7
Occupation		
Subsistence Farmer	12	40
Retired	4	13
Teacher	4	13
Pastor	4	13
Volunteer	2	7
Business	3	10
Builder	1	3

In the following, we will discuss the principal themes that were generated in the analysis of the focus groups. First, we will present overarching themes and illustrative quotes, then we will present a summary table (Table 2) that will link themes to the specific study objectives.

**Table 2:** Main themes supported by sample quotes from FGs

Objectives l	Main themes	Sub-themes	Sample Quotes
1.To determine how the cultural meaning of FGM/C has evolved over time	Theme 1: The changing culture in the practice of FGM/C	1a. Historic meaning of FGM/C	“There are those who believe that if a girl does not undergo circumcision she will not get married, that is why you see some people still continuing with this dirty practice, although nowadays people have understood that circumcision has no importance; whether one has undergone it or not they still get someone to marry them.”(TEF1, FG4).
		1b. Reasons why FGM/C persists; Evidence of continued practice	“And for me if I was to add something there, circumcision of girls ended a long time ago, but in our own community here, although we are saying there is no circumcision, there are still people who practice circumcision in secret, and we cannot guarantee that all the young girls that we see are not circumcised. I once visited a lady who is my friend and asked where the daughter was, at that same time her boy had been circumcised, and she told me that her daughter was visiting the auntie in XXX, (anonymised) (… ..) I returned to pay a visit on another day and a small girl told me, ‘auntie you were wondering where their daughter is? She is in that house there’ (*laughter from the group)*, that is probably 2 years ago(… … ….)So you see, this issue has never really gone away, it’s just that not everyone is practicing” (TEF30, FG4).
		1c. A change in culture; Evidence of a drop in the practice	“In the past, people used to say they wanted to be circumcised very much, a lot, for example like me, I offered myself to get circumcised, but later on when I got circumcised and forgot about the experience I came to realise that it is not a good thing to do, as for me two of my children are not circumcised, ehhh I realised it is not a good thing, ehhh” (TW2, FG1). “I would say it’s hard to know whether it is still happening, in the past yes when we were young children, but in my view I think this does not happen anymore, not like it used to happen in the past, I don’t think I would dare to circumcise a girl now “(TW1, FG1).
2.To assess men’s and women’s perceptions regarding the practice of FGM/C	Theme 2: The role of men and women in the continuation or ending of the practice	2a. Cultural beliefs prevail	“The reasons are the same as before, because if one is not circumcised, they are not considered ‘complete’ in the community. It is when you get circumcised that’s when you are considered a proper person, and that is when it is possible to get a man to marry you” (TW4, FG1).
		2b. Men and women may play a role in the continuation or the ending of the practice	“Sorry to say mothers or women are the ones who can easily be convinced to get their girls circumcised in this way, so even peer groups today can lead to the continuation of the practice.” (TEM 28, FG4). “But there are also some men who may not want to have their girls circumcised, but are influenced by their parents who tell them that the reason your wife does not respect you or why your children lack respect for you, it is because they are not circumcised(… … ….) this idea does not come from the man himself, its normally influence from other people outside the family, who tell the man that the reason why your wife does not respect you is because she is not circumcised.” (TW3, FG1).
		2c. Vulnerable groups to the practice	“Most of those who get circumcised do not attend school, and parents who are not in a position to educate their children may circumcise their girls. The father also can insist to their children that they must get circumcised; even when the mother insists the girl will not get circumcised.” (TW11, FG 2). “There are girls who are orphaned and although their parents belonged to the church if those who adopt them believe in the practice, they may force the orphans to get circumcised, particularly if the adopted girl starts misbehaving or spending time with boys. Those who have adopted them might say that the reason they are misbehaving is because they did not get circumcised.” (TW5, FG1). “But also grandchildren are at risk this is because if a grandchild visits the grandmother and the mother wants her to be circumcised, the grandmother can easily arrange for someone to do this! (TEM 28, FG 4)
3.To understand the effectiveness of interventions designed to fight FGM/C.	Theme 3: The impact of interventions and campaigns to end FGM/C	3a. Church, Christianity. Religious groups	“The churches, especially the Methodist church, played a big role in fighting the practice, and it was made to look like a shame to undergo the practice, and any family linked to the practice was viewed …. ai ai ai ai in a very shameful light!” (TEM 28, FG 4).
		3b. The law and prosecution; Community policing; Public meetings	“Most people got into the church; once they are taught the consequences of circumcision in church they will not return home to practice it. Again during meetings with the chief people are told to reveal those who have carried out the practice and these people then get arrested.” (TW5, FG1). “The law has helped, but very little, because in my view, if for example I take my girl and circumcise them away from my house, where is the law? I will circumcise her and she will return to the community and continue with her daily life, so what is important is working with the community itself, we need to deal with men, the girls, women, engaging directly with them and telling them about it, teaching them and showing them, that’s it, so that they can know themselves.” (TW7, FG1).
		3c. Alternative ritualistic programmes (ARPs)-*Ntanira na Migambo*	“In this area we have these groups known as *Ntanira na Migambo* and these have helped a lot, since they started a lot has changed, they teach about dangers of the practice. They come especially in December to this school near here and the girls are taught about the dangers and they are even shown a film about the practice and this has helped this area.” (TWM13, FG3). “There are parents who refused to take their children there, saying she will remain at home and not attend, even when the girls wanted to attend, and the parent refused, we see today that those who attended those programs are now good, they are well behaved, and they are educated, they went ahead with education, yes they continued with their education, because they were taught the consequences of circumcision and refused to go that route, and these are children who are respectful.” (TW2, FG1). “Some of these girls now serve as role models to others; they talk to those who did not attend such events.” (TW5, FG 1). “What I can say from my experience and from the girls that I have mentored, none of them wants to be circumcised, I have seen two girls in a family where all other girls got circumcised, these two girls did not get circumcised. The parents of these girls tried to convince them to get circumcised and what they said to them was to try and find out whether there was any difference between them and their circumcised sisters, so nowadays most girls do not want to be circumcised, they have completely refused, that is what I have personally seen. Most of them say they will not get circumcised even if it means they do not get someone to marry them.” (TEF22, FG4). “There are conferences now for both boys and girls to educate them about circumcision, pregnancy, and this has helped, if you come to my area there are no circumcised girls. Even those who are not church goers are shunning this practice. If men refuse to marry uncircumcised girls and if parents continue shunning the practice it will end.” (TWM, FG3).
		3d. Education	“You know first and foremost what we really want to eliminate is circumcision,we want them to forget that thing completely, so we would instil in them the value of education, the importance of being disciplined, to be God-fearing, and leading a good life which is Godly, and nothing else outside that. So very, very much we emphasised the importance of church and education.” (TW4, FG1). “My husband believed in educating his children and he helped me shun girls’ circumcision.” (TW11, FG2). “I think it’s even the girls themselves, education and also socialising with others and knowing that doing so (FGM/C) is not beneficial to them.” (TW7, FG2).
		3e. Health personnel	“Yes, then again health personnel, in particular public health, have been asked to talk about circumcision whenever there is a public gathering, they start teaching so that those in attendance can hear. Then again even in hospitals there is normally sensitization and teaching, the administration can plan to hold a talk with all the patients present to highlight the dangers of circumcision.” (TW3, FG 1).
4. To identify potentially effective and sustainable interventions	Theme 4: Actions perceived as most likely to bring about change	4a. Reviving and supporting ARPs	“As for us what we would like … what we would like is support so that we can invite these girls for a seminar, say for example in December when schools close, because now you have come and reminded us of what we used to do, what we were unable to carry out, if we can get someone to empower us we can organise ourselves so that say if it’s in December, we call the girls just like we used to before.” (TW2, FG1). “What I want to add, above all else is the training for advocates like us because once we get trained, we know the villages that have that problem and we can visit that particular village, we would time when they have big meetings/ gatherings, meet them first thing in the morning, because you have the knowledge and you are able to approach them, you go teach them, and anyone who wants to listen and learn will learn there and there, so that they can know there are alternative rites of passage*- Ntanira na Migambo*, because it is possible that many of them do not know, they do not attend any church, they just stay in the village.” (TW3, FG1).
		4b. Educating communities particularly young men about the practice	“It’s through education, we need to educate women about it, and also the young men, If young men took a firm stand and said “we are against FGM” and that none of these men will marry a circumcised girl, that is why you see today that young men say to their prospective wives that they want to marry an uncircumcised girl (… ……… …).” (TEF25, FG4).
		4c. Encouraging fathers’ involvement in the upbringing of their daughters	“People need more teaching and seminars in churches are desperately needed. Men also should improve their relationships with their daughters so that they can know what is happening in their lives, because as we said girls tend to confide in their mothers.” (TWM15, FG3).
		4d. Including the topic of FGM/C in the current education curriculum and public fora,	“Is it possible to work with the Ministry of Education to include this topic in the curriculum? There is no such topic covered in the curriculum at the moment other than the rites that were performed a long time ago. So this topic has not been introduced in the curriculum and it would be a good idea to incorporate in there.” (TW7, FG2).
		4e. Strengthening the community policing strategy -*Nyumba Kumi*,	“Also there are those who have been elected to be in the ‘Nyumba Kumi’, these people know what happens in every household and report any such cases. So having these people is helping people in the community live well, they are doing a great job, because if you have a visitor in the home as a member of ‘Nyumba Kumi’ I will know. If you have circumcised your daughter I will know as a member of this ‘Nyumba Kumi’.”(TW5,FG1).
		4 f. Setting up community centers for orphans.	“I think even as the community works on these issues they should pay attention to the needs of the orphans so that they can even be placed in schools for the orphans, this might help bring down circumcision and like in XXX (anonymised) there are so many children who are not in school, some are orphans and normally if one person volunteers to help such kids people see like that person is doing nothing and do not even support her, in that area there are so many children who are not in school, and circumcision will not end if these kids are not in school. For this to end there is need to target those people who have that problem, not those who already know about it.” (TW6, FG1).

### Theme 1: the changing culture in the practice of FGM/C

A pervasive theme that emerged in all four FGs was how cultural norms at various time points had operated to enable or and inhibit the practice of FGM/C. This theme explores how the cultural meaning of FGM/C has evolved over time and examines the perceived prevalence of FGM/C from the perspective of participants.

Participants described the historic meaning of, and reasons for, FGM/C for families and communities. There was widespread agreement among participants and across FGs on how the meaning and purpose of FGM/C primarily related to a girl’s suitability for marriage, and through taking part in this traditional rite of passage, a girl could be prepared and educated about how to take care of her husband and family. There was also a perception that having a child would be more challenging for uncircumcised woman, and that circumcision would make conceiving a child more likely.

“They used to say that for an uncircumcised girl, having a child would be difficult, but once circumcised this made it easy, because then she will get to be with a man, so it was mandatory.” (TWM17, FG3).

One participant stated that “in the early days, this was an important part of a girl’s life that could not be escaped” (TWM21, FG3). This participant then described the activities that typically took place during the seclusion when a girl was recovering from the procedure:

“During the circumcision seclusion period a girl would be taught how to grind traditional porridge, how to take care of the husband, care for children, and have children. This was an important part of each girl’s life, they were even taught about menstruation so that when they began their period, they would realise it was a normal physiological process of the body and not an ailment. If a girl was not part of the circumcision how would she know this?” (TWM21, FG3).

However, participants also describe how FGM/C could be used as a tool of social control, where girls and women could be forced to undergo the procedure if they were considered undisciplined, immature or lacking in self-control. Following FGM/C, those women would be considered worthy of respect and marriage, whereas “uncircumcised girls were not considered complete” (TEM28, FG4). There was a widely reported belief that women would be more likely to be “promiscuous” (TW7, FG2) and unfaithful to their husbands if they were not circumcised.

“No one will want to associate with you as a ‘mukenye’ (uncircumcised girl), your peers and other women will alienate you. Your peers will not invite you to their parties, you will not be able to engage in the activities that they do, so you are just lonely. During circumcision ceremonies they used to sing songs to mock uncircumcised girls (TWM18, FG3).

“Once a girl got circumcised she was then considered mature and this came with self-discipline, self-control, it’s like she had crossed a certain bridge, and a circumcised girl had respect.” (TWM21, FG3).

All participants noted a substantial shift in the practice of FGM/C. Many stated that they believed it did not happen at all anymore, and they were unaware of any instances in recent years. However, other participants reported that the procedure was still occurring in certain areas, but that the practice had been forced to happen in secret. The initiatives to tackle FGM/C in these communities (discussed in detail in another theme) have demonstrated considerable success and as a result, the shame and disrepute that once was associated with those who had not undergone the procedure is now very much focused on those who still practice, support or enable FGM/C. One participant reported that “it’s not like it used to happen in the past” and that they would not “dare to circumcise a girl now” (TW1, FG1). Another reported that people are “afraid” (TW5, FG1) to practice FGM/C now, due to the legal repercussions and the modern societal views on the practice.

“Most of the younger generation now is not circumcised and you hear of girls getting married all the time and they are not circumcised. And in fact, today if people know you are circumcised it is like shameful.” (TEM29, FG4).

“You will find that those who are getting circumcised are doing this in secret because it’s sort of shameful, so circumcised girls nowadays feel isolated the same way uncircumcised girls in the past used to feel.” (TWM14, FG3).

Regarding the purported prevalence of FGM/C, there was a consensus that it has drastically decreased in recent years, but again views differed on the current situation. One person estimated that there “are only about 10 per cent who are still doing it” (TEF24, FG4) but many others had only heard occasional, and increasingly rare, stories about the practice going on. There was broad agreement that the “practice has not died out completely but there has been a reduction in those that are performing it” (TEF22, FG4). Most often when the practice was reported to be occurring, it happened in secret, or it was conducted away from the girl’s local village so that it would not be known locally.

“There are those who are doing it in secret for example a girl may come home for school holidays and the parent decides that she will need to be circumcised because the parent believes that the daughter will not get married if she is not circumcised.” (TEF23, FG4).

### Theme 2: the role of men and women in the continuation or ending of the practice

A second major theme was the role of men and women in the continuation or the abandonment of the practice. There seemed to be consensus that parents generally play a crucial role in the decisions and in some instances, girls were perceived as in support of the practice. However, women, and particularly mothers, were believed to be the main support and perpetrators of the practice. Equally some participants considered grandmothers, particularly those caring for orphans and with no knowledge about the dangers of FGM/C, as more likely to have their granddaughters circumcised. Overall, views on whether it is men or women who support the continuation of the practice were mixed. Some participants saw this problem resulting from a lack of awareness and knowledge pertaining to the dangers of the practice on the part of the parents.

In terms of parental role, participants maintained that;

“So you see there is a point where parents are the ones responsible for its continuation, the girls may not want it but the parents force them because as a parent if I do not support this practice, my children will not undergo it.” (TEM28, FG4).

“Another thing, is that there are some men who normally advocate for daughters to get circumcised, because again this will depend on the knowledge that this man has, because if he still holds on to past beliefs about circumcision, a time might come, or if the girl happens to misbehave he might say ‘this girl should be circumcised.” (TW4, FG1).

In some instances, the girls themselves were viewed as wanting to undergo FGM/C.

“In the case of a girl who willingly gets circumcised; even her father will never find out. All he might be told is to contribute money to buy medication for his sick daughter but he will never know what this medication is meant for. So if you see a girl who is educated and still agrees to circumcision, then there is something unusual in this case, and this is very rare.” (TWM21, FG3).

Some of the reasons why men may encourage the practice were echoed in several FGs.

“But the other reason why a man in the family might insist on circumcision is poverty, because if he is not in a position to educate the girl he will insist that she gets circumcised, so even poverty might perpetuate the practice.” (TW7, FG 2).

However, there appeared to be consensus that women, particularly mothers and grandmothers were responsible for enabling the practice. Even among couples who agree that their daughters would not be circumcised; participants still maintained that some mothers would still have it performed covertly.

“Listen to me (to the moderator), the wife does not tell the husband that their daughter will be circumcised, the man would normally say no girl will be circumcised in this homestead and agree this with his wife, but the girl herself might want to be circumcised, listen, listen to what I am saying, so the girl wants to be circumcised and the mother wants her circumcised, so what they do is make this plan together, the girl ‘falls sick’ and becomes bedridden, she will not go to the uncle’s because it will be known what she is going to do while she is there. This plan is done in top secret because if the man were to find out he would ‘kill’ the wife*.”* (TWM18, FG3).

“To say the truth, nowadays men do not want circumcised girls. Let me be honest and say all of us men here, none of us has not had sex, and we have had sex both with circumcised and uncircumcised girls, and to say the honest truth, men of today say that if you get circumcised I will not want to be with you or have sex with you because I will not enjoy it as much as I would if you were not circumcised. And this aspect is discouraging the circumcision of girls.” (TWM21, FD3).

The role of grandmothers in the continuation of the practice was underscored in various FGs.

“But there are some grandmothers who want their granddaughters to get circumcised because I know a girl who visited her grandmother, who urged her to get circumcised, and the grandmother started calling her names because she is not circumcised, so this girl came and told me that when I visited that granny, she told me that girls should be circumcised, and then I realised that some women are encouraging girls to get circumcised.” (TW6, FG1).

“But you know the grandmother who is caring for these orphans is bringing them up just the way she knows, because for her nothing has changed, and all she has known is that circumcision is there and it is a good thing, and has importance, and she probably has never heard that people no longer circumcise.” (TW4, FG1).

There was a sense that women in these communities tend to be more influential, acting as decision makers and even taking on traditional male roles. This potentially leaves men with little room to make life-changing decisions about their daughters. Participants expressed that despite the influence of socio-cultural changes in modern society, Meru traditional culture still prevails whereby role expectations of men and women are distinct with the father taking a more authoritarian and disciplinarian role while the mothers take on a more nurturing role.

“You know once a girl attains the age of 10, she cannot have a close relationship with her father, she is closer to the mother and spends most of the time with her.” (TWM20, FG3).

“There is another mistake among the Meru people, the fact that fathers cannot get close or talk freely with their daughters once they reach a certain age, I think the big problem arises because fathers cannot talk to their daughters about this, they have to keep a distance, I think this is another big … big problem.” (TWM20, FG3).

Where there was familial and peer pressure for FGM/C, this was considered a significant enabler of the practice. Families and parents who remain traditional in their views around the importance of FGM/C to ensure a girl’s readiness for marriage were most likely to still engage in the practice. Furthermore, if some of the children in the family had been circumcised, parents may feel all children should be treated equally and therefore undergo circumcision.

Peer pressure from other girls and a desire for inclusion in social groups was also cited as a potential enabler of FGM/C.

“One of the reasons why the practice continues is the parents, although the culture is changing they do not want to have some of their children circumcised and others not circumcised. They want all children to be the same. The other reason is social groups, if most of the girl’s peers are circumcised then she might also feel pressurised in order that she is accepted in that group.” (TEM26, FG4).

### Theme 3: the impact of interventions and campaigns to end FGM/C

The third major theme highlighted the effectiveness of interventions designed to fight FGM/C. There was agreement in all four FGs that both the church and education had a significant impact in shifting the culture so that FGM/C was no longer normalised. Those who did not attend church or those who do not send their children to school were identified by participants as families most likely to still be engaged in FGM/C. In fact, a child being in school was considered both a preventative and a protective factor, because “there is no time to circumcise her” (TWM16, FG3).

“We have put in a lot of effort in terms of teaching people about circumcision and the dangers of it. It reached a point where if a woman was known to have circumcised her daughter she was excluded from the church, and this led to many of them performing the practice in top secrecy” (TEF, FG4).

“Those who mostly circumcise their girls are those people who are not Christians, people who do not go to church. The husband and wife do not go to church and hence do not know the negative consequences of such a practice and most of these teachings have come from the church.” (TW10, FG2).

As a result of receiving education on the dangers of FGM/C in schools and in the church, there was a perception that girls and young women are better equipped to resist the practice. Having positive “role models” (TW5, FG1) in the community who were open about not undergoing FGM/C also enabled peer support and reduced any feelings of isolation or being seen as different.

“Education still plays an important role in ending circumcision because girls who are educated know their rights … You know when a girl goes to secondary schools and knows that other girls are not circumcised, and the teachers are also not circumcised, and their lives are alright, this helps a lot.” (TWM21, FG3).

“Nowadays most girls do not want to be circumcised, they have completely refused, and that is what I have personally seen. Most of them say they will not get circumcised even if it means they do not get someone to marry them.” (TEF22, FG4).

Where men’s desires to maintain control over a woman and ensure she was ‘disciplined’ once encouraged the practice, the education of men on this issue had supported this cultural shift to the extent that men do not want to marry circumcised women.

“Even the men of today; they do not want circumcised girls; and this means that there is the potential for girls’ circumcision to die out completely.” (TWM20, FG3).

The practice of FGM/C was outlawed initially in the early 1960s followed by newer legislation in 2011.While the law was considered instrumental in initiating change, participants noted that it is the church and education in schools that laid the groundwork and shifted the norms sufficiently such that when the new law was introduced, it was effective in this new context where FGM/C had taken on negative connotations. Together, these changes resulted in a considerable change in the culture to one that is almost the antithesis of the culture 20–30 years ago, where the practice FGM/C was mandatory.

“Let us tell you two things that make the practice go under: The law and Christianity. Because for me as a pastor, a member of my congregation will not want me to know that he or she has circumcised their daughter. This is something that was happening long before the law was put in place.” (TWM21, FG3).

“The reason why the law was not effective then is because people were not as educated as they are today. I would say education is what has led to the decrease in the practice, because boys got educated and girls as well, and the girls got to know their rights.” (TWM14, FG3).

Finally, participants highlighted that community policing strategies, such as the *Nyumba Kumi*^1^initiative, introduced in the country between 2007 and 2008, may have played a part in changing norms regarding the practice of FGM/C*. Nyumba Kumi* is a strategy of anchoring community policing at the household level and is aimed at bringing the local community together in a pursuit of common ideals such as a safe, sustainable and prosperous neighbourhood. Some FGs participants reported that this strategy operated in villages to monitor and report when the practice was occurring so that those responsible could face consequences.

“There are those who have been elected to be in the *Nyumba Kumi*, these people know what happens in every household and report any such cases. So, having these people is helping people in the community live well, they are doing a great job, because if you have a visitor in the home as a member of *Nyumba Kumi* I will know. If you have circumcised your daughter I will know as a member of this *Nyumba Kumi*” (TW5, FG1).

### Theme 4: actions perceived as most likely to bring about change

The fourth theme was around actions that are likely to bring change in the practice of FGM/C. Participants described initiatives and actions such as 1) reviving and supporting ARP initiatives, 2) encouraging fathers’ involvement in the upbringing of their daughters,

3) inclusion of the topic of FGM/C in the current education curriculum and public fora, 4) strengthening the community policing strategy -*Nyumba Kumi*, 5) setting up community centers for orphans.

#### Reviving ARP initiatives

Participants also felt that ARPs such as *Ntanira na Migambo* (circumcision through words) that existed in the past should be revived and organised at the grassroots level. These groups were organised during school holidays and supported by non-governmental organizations such as Plan International. Every year at least 200 girls were trained, and the expectation was that once these girls left the training they would go and tell others what they had learned. Every year the number of girls who attended grew bigger in numbers, but eventually as funds run out the advocates were unable to continue with the program.

“When we had started such a campaign we had received support from an organization called Plan International, they are the ones who started such a campaign, inviting us to attend a one week seminar, as leaders, which was in 2007/8. They had taken leaders of different churches, 2-3 people from each church, and these people would then bring back the message to the church, and at this point the church would then organize and see to it that the campaign against circumcision took place. There was also another group that came in called ‘Good Samaritan’, I think these are the two groups that supported us.” (TW4, FG1).

“We lacked the finances, and then realised we cannot be able to support the girls as a group the way we used to, so we decided to continue teaching in the churches and at the grassroots level, if possible we teach them ourselves or find for them teachers to do that in the churches, like during youth seminars, something like that.” (TW7, FG2).

Participants also underscored the need for more training and financial supports to enable them carry out sensitization activities in the communities where the practice is still on-going.

“What I want to add, above all else is the training for advocates like us because once we get trained, we know the villages that have that problem and we can visit that particular village, we would time when they have they big meetings/ gatherings, meet them first thing in the morning, because you have the knowledge and you are able to approach them, you go teach them, and anyone who wants to listen and learn will learn there and there, so that they can know there are alternative rites of passage- *Ntanira na Migambo*, because it is possible that many of them do not know, they do not attend any church, they just stay in the village.” (TW3, FG1).

“If we organize a group and this group gets support, they can go out and teach others, because, say for advocates like me, it is difficult to cater for my expenses to travel to areas like XXX (anonymised) or XXX (anonymised) where I know there is sensitization needed. I would require money to cater for my transportation. Again you would not go to talk to those people without support, they would not listen to you.” (TW5, FG1)

#### Encouraging fathers’ involvement in their daughters lives

Men were generally deemed as opposed to the practice and hence were considered as influencers in stopping the practice. Participants, particularly male participants, underscored the lack of close relationships with their daughters as a potential deterrent to realising change in the practice. Most noted that if men were more closely involved in the upbringing of their daughters, they would most probably be privy to any plans to have their daughters subjected to the practice and would be more influential in effecting change.

“What I can say is that today women are the ones with the most responsibilities in the home, they are influential in the home, even, you go and check the road constructions that are being carried out and you will see most of the casual work is being done by women, if this is the case how will the women not yield more power. Men ought to be more responsible especially this younger generation that is how we can finish this. If they do not take responsibility the women will overrule them. (TWM18, FG3).

“I think that the problem is that men in the traditional Meru family have not been actively involved, I think if they were involved the way women have been, this practice would have ended, and actually the wife may be afraid to subject their daughters to this if the men in the families were well aware of what goes on. Men have not been well involved.” (TEM 29, FG4).

#### Including FGM/C as a topic in the current school curriculum and in public discourses

The need to include FGM/C as a topic in the current school curriculum and openly discuss FGM/C in public forums and parliamentary sessions was suggested.

“Is it possible to work with the Ministry of Education to include this topic in the curriculum? There is no such topic covered in the curriculum at the moment other than the rites that were performed a long time ago. So this topic has not been introduced in the curriculum and it would be a good idea to incorporate in there. I suggest we include the teachings in the school curriculum and there should be forums to talk about the practice and also parliament should debate this issue thoroughly, may be this would make it more effective.” (TW7, FG2).

#### Building on existing community-based strategies

Participants argued for the need to adopt a bottom-up approach in dealing with the practice as they considered this to be the most effective avenue to eliminate the practice. In particular building on systems already in place, such as the *Nyumba Kumi* strategy that focuses on information sharing, especially over security threats was believed would be hugely effective. While participants also underscored the shortcomings of this community policing, there was consensus that if strengthened it would be the most effective and potentially the most sustainable to help determine households that were still practicing FGM/C and girls that are vulnerable.

“I think what I would like to add is that I do not think dealing with the issue from the very top is as effective as starting from the grassroots level, so you know, if we can involve the *Nyumba Kumi* they might be instrumental in helping stop this practice, because they will know whether or not the practice is going on.”(TW10,FG2).

#### Setting up community centres for orphans

There was consensus among participants of the need to pay particular attention to the needs of orphans and placing them in schools or community centres. This it was believed would potentially reduce the incidences of FGM/C in this area. Orphans were described as the most vulnerable to the practice of FGM/C with orphaned girls more likely to get married off early.

“There are girls who are orphaned and although their parents belonged to the church if those who adopt them believe in the practice, they may force the orphans to get circumcised, particularly if the adopted girl starts misbehaving or spending time with boys.” (TW5, FG1).

“I think even as the community works on these issues they should pay attention to the needs of the orphans so that they can even be placed in schools for the orphans, this might help bring down circumcision and like in XXX (anonymised) there are so many children who are not in school, some are orphans and normally if one person volunteers to help such kids people see like that person is doing nothing and do not even support her. In that area there are so many children who are not in school, and circumcision will not end if these kids are not in school. For this to end there is need to target those people who have that problem, not those who already know about it.” (TW6, FG1).

## Discussion

The practice of FGM/C remains prevalent across Africa. Kenya has comparatively lower rates than many other countries but demonstrates substantial variations among counties within the country. A spatial modelling study highlighted the continued high prevalence clusters of FGM/C in North Eastern and South Western Kenya [19]. The present qualitative study was the first to review perceived recent changes and factors that are responsible for the persistence of FGM/C in two rural areas of Kenya from the perspective of activists. Guided by a context-focused framework analysis [18] we found that the perceived meaning and cultural significance of FGM/C has changed over the years in the rural setting of our study. Beliefs that girls who were not circumcised would not find a husband are not common anymore. In fact, the FG participants pointed out that the generation of their daughters and granddaughters was less likely to have undergone FGM/C. At the same time, they pointed out that some continue the practice, mostly covertly.

Peer pressure, including a desire for inclusion in social groups, seems to be a potential enabler of FGM/C in this context. The evolution of commonly held interdependent, collective beliefs in communities, i.e. ‘social norms’, is seen as potentially effective as a first step in achieving sustainable behaviour change. Social norms theory has informed the development of interventions targeting the abandonment of FGM/C. A recent study in Senegal and Gambia identified in focus groups several normative pressures, including ‘ostracisation of uncut women’; ‘peer pressure among girls’; ‘proper parenting’; ‘moral virtue’ with considerable consensus among older and younger women but less so between regions. The study also showed that maintaining FGM/C is very much linked to the view of maintaining traditions and that older women demonstrated the greatest ambivalence towards abandonment, while younger women were less likely to advocate for abandonment of the practice. Younger women were most conservative, while older women, while being custodians of traditions, demonstrated greater openness towards change [20]. In our study, grandmothers in particular were identified as influential in perpetuating the practice due to the perceived value of upholding traditional practices and their authority in these communities. However, recent research [20] has highlighted the potential role for grandmothers in challenging and contesting the practice and in so doing, being effective agents for supporting cultural change. This suggests that older women are influential in the community and valuable targets for interventions to support abandonment of the practice.

Poverty was cited as another reason why families in this context continue to perform FGM/C. The relationship between poverty and FGM/C remains inconclusive. A recent Nigerian study found no direct or simple relationship between poverty and FGM/C [21]. It is difficult to disentangle the relative contribution of poverty from a complex bundle of risk factors that also include low education, literacy and cultural factors [22]. It is important to address the factors in the complex causation bundle, which are more readily amenable to change. Foremostly, education in schools to strengthen literacy, work with cultural leaders (chiefs), and engagement of religious organizations should be a focus of future interventions.

There were sentiments, expressed, mostly by male participants, that women, particularly mothers had a role to play in the continuation of FGM/C in this community. The participants attributed this to the shifting gender relations in the region whereby some women now have more decision-making power and are increasingly becoming financially independent [23]. It was asserted that being in such a position potentially gives women the power to make decisions about the welfare of the children, without involving their partners. Kenya’s 2010 constitution recognises the role of women as key players in the political and social-economic sphere, which has led to an increase in women’s participation in the workforce, and subsequent financial contributions to the household [24, 25]. Previously men’s identities as sole breadwinners gave them immense control over decision-making within their families; an authority that is increasingly being undermined by their wives [25]. Rapidly shifting gender relations made men in this study unsure about their authority to challenge and firmly oppose the practice of FGM/C within the family.

Yet, some participants viewed men as potentially powerful allies in the effort to end FGM/C in our study. This was also found in a recent peer-led exploration of young people’s attitudes towards the practice in a community in Eastern Kenya with a particularly high prevalence rate [26]. In the study, most young men reported a “modern” understanding of the issue and said they would rather marry “educated” women and are opposed to FGM/C but the study reported that men found it challenging to oppose the practice. There is a need to explore and strengthen opportunities for dialogue about the practice between men and women and inform the development of strategies to address FGM/C. Valuable lessons about the most effective ways to involve men and women to work collaboratively in the SSA can be learned from women’s health programmes that involve men as partners [27].

Places of worship such as churches were perceived as powerful organisations that have contributed to the reduction of FGM/C, both as respected institutions providing moral guidance and as sites of education for the public. This is consistent with findings from Eastern Ethiopia and Egypt where study participants reported that churches and mosques provided teachings and information condemning the practice [28]. Other studies have found that interventions that place a greater emphasis on religious interpretation of FGM/C’s undesirability, are more likely to be effective, compared to those that focus on highlighting health complications or FGM/C as a violation of human rights [29]. Overall, gaining support and commitment from religious leaders is considered key to the success of FGM/C interventions.

Education appears to be a key factor in the reduction of FGM/C. The role of education has been highlighted in a number of publications on FGM/C from a variety of countries, consistently pointing to the fact that girls with no or little education are more likely to undergo the procedure and are also more likely to endorse it [21]. The level of education was also found predictive of male preferences for circumcised girls in a study from Ghana [22]. In our study participants highlighted the need to discuss the practice as part of the curriculum, especially when girls and boys are taught together. Another Kenyan study conducted among the Kuria and Kisii communities reported the need for anti-FGM/C partners to work more closely with teachers to help build their capacity and confidence to discuss the issue openly with their pupils in a safe environment [30].

As study participants highlighted in the interviews, some initiatives to end FGM/C, such as ARPs are not continued due to lack of resources and sustained involvement of local and international NGOs and faith-based organisations implementing these initiatives. Most of these programmes are small in scale and receive time-limited support or no financial or technical support from the national government making them unsustainable [14]. Governments in most countries are either silent on the issue or leave the responsibility of eliminating FGM/C to NGOs. In November 2019, Kenya’s president reiterated the country’s commitment to “...... eliminate female genital mutilation by 2022,” through the strengthening of coordination mechanisms and by addressing cultural norms that propagate these practices,” However, without sustainable funding particularly for grassroot organisations, this ambitious target is unlikely to be met. Coupled with the need to allocate more funding to anti-FGM/C interventions, it is crucial that adequate funding be allocated to researchers at local universities that would enable the conduct of research to inform implementation of effective and potentially sustainable interventions.

We also suggest that future interventions conduct a form of ‘diagnosis’ of readiness to change and then a whole systems approach tailored to the specific circumstances of communities. Matanda and colleagues in their recent work [31] highlight the need for greater emphasis on complex, systemic factors in designing interventions to abolish the practice, while pointing to substantial socio-demographic and geographic variability. Simple interventions are unlikely to bring about change in a practice that is maintained by complex social norms. Systems vary locally and overtime. It is important to identify the reinforcing (maintaining the status quo) and balancing factors (limiting or mitigating circumstances).

Our study had some limitations; resource constraints precluded a wider exploration of FGM/C practices across Kenya. While FGM/C rates vary across Kenya [32] some of the areas with the highest practice prevalence rates could not be included in the study. Insights from advocates involved in this study may not be transferable to those regions. The present study only focused on the perspectives of senior anti-FGM/C activists, and did not include other voices, notably those of girls and women, men, community leaders, legislators and law enforcement. Despite these limitations, the study has revealed important factors that contribute to the continuation of FGM/C in rural areas as well as potentially underutilised strategies and actors to limit the practice.

## Conclusion

Our study shows that current and past efforts to tackle FGM/C are clearly not working. It would appear that efforts to tackle the practice have been highly fragmented and not followed a whole system approach. While the practice of FGM/C is illegal in Kenya our study suggests that it is still being conducted in secret in some communities such as Tigania and Igembe in Meru county. Our study reiterates the role that ARPs can play in sensitizing communities about the practice and in educating girls, in particular. In Meru county activists have been at the forefront in leading ARPs, but their work has been hampered by a lack of finances, training and support from the county government and NGOs. And while ARPs have been rather successful, they are not a one-size-fits all intervention.

Clearly, contextualised, comprehensive approaches are needed that combine comprehensive education at primary, secondary and adult education levels, attention to the risk of orphans to be exposed to the practice, the support of religious leaders, women as role models and men gaining a voice in the debate to advocate openly for the abandonment of the practice. It will require a combination of behaviour change support at the community level, law enforcement and monitoring, and open and persistent advocacy by diverse representatives of communities.

## Data Availability

The data analyzed for this manuscript are available from the corresponding author on request.

## Abbreviations

ARPs: Alternative ritualistic programmes
FGM/C: Female Genital Mutilation/Cutting
FGs: Focus Groups
MYWO: Maendeleo ya Wanawake Organization
NGOs: Non Governmental Organisations
PATH: Program for Appropriate Technology in Health
SSA: Sub-Saharan Africa

## Footnotes

1 Community policing strategy

## References

[1] WHO. Female genital mutilation 2018. https://www.who.int/news-room/fact- sheets/detail/female-genital-mutilation. Accessed 11 Apr 2019.

[2] UNICEF. Female genital mutilation/cutting: a global concern [press release]. 2016. https://www.unicef.org/media/media_90033.html. Accessed 4 Sept 2017.

[3] Johansen REB, Diop NJ, Laverack G, Leye E. What Works and What Does Not: A Discussion of Popular Approaches for the Abandonment of Female Genital Mutilation. Obstet Gynecol Int. 2013. 10.1155/2013/348248. 10.1155/2013/348248 PMC3655658 23737795

[4] Denison E, Berg RC, Lewin S, Fretheim, A. Effectiveness of interventions designed to reduce the prevalence of female genital mutilation/cutting. Report from Kunnskapssenteret Nr 25–2009. https://www.fhi.no/globalassets/dokumenterfiler/rapporter/2009-og-eldre/rapport_0925__fgm_kjonnslemlestelse.pdf. . 29320117

[5] Toubia NF, Sharief EH (2003). Female genital mutilation: have we made progress?. Int J Gynecol Obstet.

[6] Chege JN, Askew I, Liku J. An assessment of the alternative rites approach for encouraging abandonment of female genital mutilation in Kenya. US Agency for International Development. 2001. doi:10.31899/rh1.1009. Accessed 6 Feb 2016.

[7] Graamans EP, Zolnikov TR, Smet E, Nguura PN, Leshore LC, Ten Have S. Lessons learned from implementing alternative rites in the fight against female genital mutilation/cutting. Pan Afr Med J. 2019;32. 10.11604/pamj.2019.32.59.17624. 10.11604/pamj.2019.32.59.17624 PMC6560975 31223351

[8] Kenya Bureau of Statistics-Government of Kenya. Demographic and Health Survey 2014. Rockfield, MD USA 2016 https://dhsprogramcom/publications/publication-fr308-dhs-final-reportscfm Accessed 11 Mar 2017.

[9] Thomas L, Shell-Duncan B, Hernlund Y (2000). "Ngaitana (I will circumcise myself)": lessons from colonial campaigns to ban excision in Meru, Kenya. Female circumcision in Africa: culture, controversy and change.

[10] Muchui, D. War against female circumcision bearing fruit in Meru. [Daily Nation Newspaper]. 2015.http://www.nation.co.ke/counties/meru/War-against-female-circumcision-Meru/1183302-3008376-1kp3ez/index.html. Accessed 11 Jan 2017.

[11] Muchui, D. Girl died after FGM procedure; medics say. [Daily Nation Newspaper].2019. https://www.nation.co.ke/counties/meru/Girl-died-after-FGM-procedure/1183302-4996162-6bslrx/index.html. Accessed 11 April 2019.

[12] Njeru, A. Tough war in bid to end FGM in Meru. [Daily Nation Newspaper].2018. https://www.nation.co.ke/counties/meru/Tough-war-in-fight-against-FGM-Meru/1183302-4902434-15ltr01z/index.html. Accessed 11 Apr 2019.

[13] Evelia H, Sheikh M, Askew I. Contributing towards efforts to abandon Female Genital Mutilation/Cutting in Kenya: A situational analysis. Nairobi: Ministry of Gender, Sports, Culture and Social Services, Republic of Kenya.2007. https://pdfs.semanticscholar.org/7e64/2619041c4e3bfa0551ba401825b0d68c4087.pdf Accessed 16 Nov 2016.

[14] WHO. Female Genital Mutilation programmes to date: what works and what doesn’t. [Policy Brief]. 2011. https://apps.who.int/iris/bitstream/handle/10665/75195/WHO_RHR_11.36_eng.pdf?sequence=1. Accessed 6 June 2016.

[15] Murithi K. Shun FGM, First Lady Urges Meru Residents. [The Star newspaper, Nairobi] 2015. https://www.the-star.co.ke/counties/north-eastern/2015-05-29-shun-fgm-first-lady-urges-meru-residents/. Accessed 1 June 2015.

[16] Kitzinger J (1995). Qualitative research. Introducing focus groups. BMJ..

[17] Braun V, Clarke V (2006). Using thematic analysis in psychology. Qual Res Psychol.

[18] Srivastava A, Thomson SB (2009). Framework analysis: a qualitative methodology for applied policy research. J Adm Gov.

[19] Achia TNO (2014). Spatial modelling and mapping of female genital mutilation in Kenya. BMC Public Health.

[20] Shell-Duncan B, Moreau A, Wander K, Smith S. The role of older women in contesting norms associated with female genital mutilation/cutting in Senegambia: a factorial focus group analysis. PLoS One. 2018;13. 10.1371/journal.pone.0199217. 10.1371/journal.pone.0199217 PMC6059403 30044770

[21] Akindola RB, Abiola MO (2019). Is female circumcision driven by culture or poverty? Evidence from indigenes of Ikole, Oye and Ido-Osi local government areas of Ekiti state, Nigeria. Open J Soc Sci.

[22] Klouman E, Manongi R, Klepp K-I (2005). Self-reported and observed female genital cutting in rural Tanzania: associated demographic factors, HIV and sexually transmitted infections. Tropical Med Int Health.

[23] Karmebäck VN, Wairore JN, Jirström M, Nyberg G (2015). Assessing gender roles in a changing landscape: diversified agro-pastoralism in drylands of west Pokot, Kenya. Pastoralism.

[24] Ambasa C, Workneh, Musahara (2016). A holistic strategy for improving gender-power relations and food security in Tigania, Meru county Kenya. Innovations in Achieving Sustainable Food Security in Eastern and Southern Africa, Ethiopia: Organisation for social science research in Eastern and Southern Africa.

[25] Withers M, Dworkin SL, Zakaras JM, Onono M, Oyier B, Cohen CR (2015). ‘Women now wear trousers’: men’s perceptions of family planning in the context of changing gender relations in western Kenya. Culture Health Sexuality.

[26] Brown E, Mwangi-Powell F, Jerotich M, le May V (2016). Female genital mutilation in Kenya: are young men allies in social change programmes?. Reproductive Health Matters.

[27] Wegner MN, Landry E, Wilkinson D, Tzanis J (1998). Men as Partners in Reproductive Health: from issues to action. Int Fam Plan Perspect.

[28] Gele AA, Bø BP, Sundby J. Attitudes toward female circumcision among men and women in two districts in Somalia: is it time to rethink our eradication strategy in Somalia? Obstet Gynecol Int. 2013. 10.1155/2013/312734. 10.1155/2013/312734 PMC3654358 23710186

[29] Berg RC, Denison EM (2013). A realist synthesis of controlled studies to determine the effectiveness of interventions to prevent genital cutting of girls. Paediatr Int Child Health.

[30] Oloo H, Wanjiru M, Newell-Jones K (2011). Female Genital Mutilation practices in Kenya: The role of Alternative Rites of Passage- A case study of Kisii and Kuria districts.

[31] Matanda D, Okondo C, Kabiru C, Shell-Duncan B. Tracing change in female genital mutilation/cutting: shifting norms and practices among communities in Narok and Kisii counties, Kenya [working paper]. Population Council. 2019. 10.31899/rh6.1037.

[32] Kandala N-B, Kinyoki D, Sarki A, Gathara D, Komba P, Shell-Duncan B. Modeling and mapping of girls’ female genital mutilation/cutting (FGM/C) in the context of economic, social, and regional disparities: Kenya demographic and health surveys 1998-2014. Reprod Health. 2017. 10.31899/rh7.1034.

